# TD-ESI-MS/MS for High-Throughput Screening of 13 Common Drugs and 4 Etomidate Analogs in Hair: Method Validation and Forensic Applications

**DOI:** 10.3390/toxics13050329

**Published:** 2025-04-23

**Authors:** Meng Li, Jinbo Li, Binling Zhu

**Affiliations:** Department of Forensic Science, Fujian Police College, Fuzhou 350007, China; limeng@fjpsc.edu.cn (M.L.); lijinbo@fjpsc.edu.cn (J.L.)

**Keywords:** drugs of abuse, high-throughput screening, TD-ESI-MS/MS, etomidate analogs, forensic applications

## Abstract

This study established a dual analytical workflow integrating thermal desorption–electrospray ionization–tandem mass spectrometry (TD-ESI-MS/MS) for rapid qualitative screening and ultra-performance liquid chromatography–tandem mass spectrometry (UPLC-MS/MS) for confirmatory quantification of 17 psychoactive substances and metabolites across six classes (opioids, amphetamine-type stimulants, cocaine, ketamine-type drugs, cannabinoids, and etomidate analogs) in hair matrices. Validation of the TD-ESI-MS/MS method demonstrated its sensitivity (limits of detection: 0.1–0.2 ng/mg) and precision (<19.3%), with matrix effects controlled to <19.6%. The TD-ESI-MS/MS method achieved an analysis time of 1 min per sample, enabling high-throughput screening with a sensitivity >85.7% and a specificity >89.7% for the 17 analytes. UPLC-MS/MS confirmation validated the screening results with accuracy rates of 89.7–99.8%. An analysis of specimens confirmed positive identified etomidate analogs as the predominant psychoactive substances (73.6%), with a lower prevalence of amphetamine-type stimulants (12.5%), ketamine-type drugs (9.0%), and opioids (2.8%). The polydrug use patterns identified concurrent etomidate–amphetamine consumption (*n* = 5) and complex analog combinations (etomidate–isopropoxate–metomidate, *n* = 13), suggesting evolving abuse trends. Despite limitations in the temporal resolution and representativeness of the cohort, this study demonstrated the viability of TD-ESI-MS/MS for bridging forensic and public health priorities. Future work should focus on optimizing the durability of the ion source for TD-ESI and validating this method across diverse populations to enhance its generalizability.

## 1. Introduction

The global drug landscape has undergone a seismic shift with the proliferation of new psychoactive substances (NPSs), particularly synthetic analogs designed to circumvent the existing regulatory frameworks. As reported in the UNODC World Drug Report 2024, global authorities identified 1228 NPSs [1], with etomidate analogs (etomidate, metomidate, and isopropoxate) emerging as a critical public health threat [2]. Etomidate analogs have often been adulterated into e-liquids, driven by their low cost, ease of synthesis, and potent dissociative effects [3,4,5]. Unlike traditional opioids or stimulants, these analogs have evaded conventional immunoassay detection due to their structural modifications, necessitating advanced analytical methods for their accurate identification [6].

The rise of NPSs underscores the limitations of the traditional biological matrices (urine, blood) used for drug monitoring. Urine testing, while rapid, suffers from a narrow detection window (2–7 days) and susceptibility to adulteration [7]. Blood analyses, though precise, are invasive and impractical for large-scale screening [8]. These challenges have been exacerbated by the increasing prevalence of polydrug use, where users combine stimulants with sedatives to potentiate their psychoactive effects, complicating toxicological interpretations [9,10].

Hair emerged as a standard matrix for long-term drug monitoring, offering a detection window extending up to several months depending on hair length [11]. Unlike ephemeral bodily fluids, drugs and metabolites are incorporated into the hair shaft via bloodstream diffusion during follicular growth, where they bind irreversibly to keratinized proteins [12]. This process creates a chronological record of drug exposure, enabling a retrospective analysis through segmental hair testing [13]. Previous studies utilizing ambient ionization mass spectrometry (MS) for hair analysis have focused on traditional drugs but have lacked comprehensive validation for structurally diverse NPSs such as etomidate analogs [14,15]. While liquid chromatography (LC)–MS/MS has remained the gold standard for confirmatory analyses, its time-intensive workflows (≥10 min/sample) limit its scalability for high-throughput forensic scenarios [16,17]. Additionally, the existing methods rarely integrate rapid screening with confirmatory quantification into a single workflow, creating operational inefficiencies.

This study was designed to establish a dual-platform workflow combining thermal desorption electrospray ionization (TD-ESI)–MS/MS and ultra-performance (UP)LC-MS/MS for detecting six drug classes (opioids, amphetamine-type stimulants, cocaine, ketamine-type drugs, cannabinoids, and etomidate analogs) in hair. This method addressed a critical forensic need for harmonized high-throughput screening and validation, particularly for structurally modified NPSs that evade conventional detection methods.

## 2. Materials and Methods

### 2.1. Chemicals and Reagents

Certified reference materials for the 17 analytes (6-Monoacetylmorphine (6MAM), ketamine (KET), amphetamine (AMP), norketamine (NorKET), 3,4-methylenedioxyamphetamine (MDA), 3,4-methylenedioxymethamphetamine (MDMA), methamphetamine (METH), benzoylecgonine (BZE), tramadol (TRA), Δ^9^-tetrahydrocannabinol (THC), cocaine (COC), fentanyl (FENT), morphine (MOR), etomidate (ETO), metomidate (METO), isopropoxate (ISP), and etomidate acid (ETA)) were procured from Yuansi Standard Science (Shanghai, China). LC-MS-grade methanol, acetonitrile, and ammonium formate were sourced from Fisher Scientific (Waltham, MA, USA).

### 2.2. Standard Solution

Primary stock solutions (1 mg/mL) of all of the analytes were prepared in methanol and stored at 4 °C. Serial dilutions generated working solutions spanning 0.02–12.5 ng/mg, with calibration standards prepared in a blank hair matrix to match the biological specimens.

### 2.3. Sample Preparation

Hair samples and questionnaires were collected from 670 participants through collaboration with drug rehabilitation facilities and public security departments. The study population included individuals undergoing supervised detoxification programs and suspected active drug users identified during law enforcement screenings. The hair samples (segments 3 cm proximal to the scalp) were collected following the standard decontamination protocols and stored at −20 °C. Participation was voluntary and anonymized, with written informed consent obtained under institutional ethical approval.

To minimize external contamination, the hair samples were sequentially washed with 5 mL of ethanol and deionized water. Proximal segments (0–3 cm from the root) were selected for the analysis to represent their drug exposure within the past 3 months, assuming a conservative growth rate of 1 cm/month [10]. Distal segments (>3 cm) and visibly damaged/dyed portions were excluded. All of the washed hair was air-dried, cryogenically pulverized (−40 °C, 70 Hz, 3 min), extracted using methanol:water (1:1, *v*/*v*), and filtered (0.22 μm) prior to the analysis. The resulting samples were subjected to rapid screening for the 17 analytes using TD-ESI-MS/MS, which were then identified using UPLC-MS/MS.

### 2.4. Instrumentation Conditions

A TD-ESI source (Mindu Innovat Lab, Fuzhou, China) coupled with a tandem mass spectrometer (Agilent, Santa Clara, CA, USA) was operated in MRM mode. The blank and case human hair samples were then analyzed using UPLC-MS/MS (TSQ Altis Plus, Thermo Scientific, Waltham, MA, USA). The instruments’ parameters for TD-ESI-MS/MS and UPLC-MS/MS are shown in Tables S1 and S2, respectively.

### 2.5. Method Validation

Validation followed ANSI/ASB 036 Standard, the ICH Q2(R1) guidelines, and previous studies [18,19,20]. Selectivity was confirmed via a blank matrix analysis, while the sensitivity parameters (LOD: S/N ≥ 3; LLOQ: S/N ≥ 10) were established across three concentration levels (low, medium, and high concentrations). The intra-/inter-day precision and accuracy were evaluated using six replicates over six days. Matrix effects were quantified via post-extraction spiking. To minimize potential carryover effects between samples, blank sample injections were performed after every ten samples during the TD-ESI-MS/MS analysis. No detectable signals for the target analytes were observed in the blank runs, confirming the absence of carryover interference.

## 3. Results and Discussion

### 3.1. Validation of the TD-ESI-MS/MS Method for Qualitative Screening

Systematic optimization of the mass spectrometric parameters enhanced the detection reliability. A full-scan analysis (50–500 *m*/*z*) identified the precursor ions, followed by product ion selection via SIM mode. The fragmentor voltages (50–200 eV) and collision energies (5–70 eV) were iteratively optimized in MRM mode to maximize the sensitivity. The final parameters (Table S3) achieved baseline separation of all of the analytes.

The specificity of the method was validated by comparing blank hair samples with those spiked at 2.5 ng/mg. No endogenous interference co-eluting with the 17 analytes was observed at their ion transitions. Analytical precision was confirmed through six consecutive injections of spiked calibrator samples, demonstrating consistent peak intensities. The selectivity was verified further according to the absence of interfering peaks in blank hair samples to which 2.5 ng/mg of the opioids, amphetamine-type stimulants (ATSs), cocaine, ketamine-type, cannabinoids, and etomidate analogs was added (Figure S1).

The reproducibility of the method was validated through intra- and inter-day studies demonstrating its precision and accuracy across three spike levels (0.5–12.5 ng/mg) (Table 1). Matrix effects remained within 0.8–19.6% for all of the analytes, consistent with the LC-MS/MS performance benchmarks for hair analysis [21,22]. Optimization of the sensitivity using dual diagnostic ions (S/N ≥ 3) achieved LODs of 0.1 ng/mg for 14 of the analytes, while TRA, MDA, and ETA required 0.2 ng/mg due to the limitations of ionization. The method met the Society of Hair Testing (SoHT) decision thresholds (0.2 ng/mg) for regulated substances and reliably detected the emerging compound ETO at this forensic-relevant level. Combining validated specificity, analytical stability, and an ultra-rapid throughput (60 s/sample), this workflow enabled high-throughput screening of complex hair matrices for forensic applications.

### 3.2. Confirmatory Validation Using UPLC-MS/MS

A validated UPLC-MS/MS method was developed for confirmatory analysis of the 17 psychoactive substances and metabolites (Table S4). Chromatographic separation was achieved within 10 min using a C18 column, resolving structurally analogous compounds at the baseline.

The method achieved a sensitivity constituting LODs of 0.01–0.02 ng/mg and LLOQs of 0.02–0.05 ng/mg (Table 2), below the thresholds recommended by the SoHT (0.2 ng/mg for most regulated drugs). Although TRA, MDA, and ETA exhibited slightly higher LOD/LLOQ values (0.02/0.05 ng/mg), likely due to ion suppression [23], their LODs remained forensically actionable for detecting chronic abuse patterns. The calibration curves demonstrated linearity across 0.02–5.00 ng/mg (R^2^ ≥ 0.9953, weighted 1/x regression). The intra-day accuracy (1.1–8.9%) and inter-day precision (<9.4%) validated the method’s robustness, complying with the ANSI/ASB 036 standards for forensic assays [24]. The LLOQ of 0.02 ng/mg reliably identified etomidate analog exposure, critical for monitoring emerging e-liquid adulteration [3].

Previous studies [22,25,26,27,28] have reported the detection and analysis of drugs in hair samples using LC–MS/MS and gas chromatography (GC)–MS/MS. For instance, high-speed bead grinding coupled with LC–MS/MS was able to simultaneously quantify 21 drugs (amphetamines, opioids, and cannabinoids) within 20 min, demonstrating a LOD of 0.01 ng/mg [27]. Peng et al. reported that LC–MS/MS was able to identify quetiapine with a sensitivity of 0.15 ng/mg [26]. Derivatization-enhanced LC–MS/MS precisely quantified propofol metabolites at 3.6–7.8 pg/mg [25]. A hybrid GC–MS and LC–MS/MS method was able to detect imidazole-derived GABA agonists with detection limits spanning 0.0591–130 ng/mg within 12 min [28]. A Box–Behnken response surface methodology enabled an LC–MS/MS analysis of nine fentanyl drugs within a concentration range of 3.0–220.0 pg/mg [22].

### 3.3. Practical Forensic Applications

#### 3.3.1. A Case Study: Concurrent Screening and Confirmation

**Figure 1:**
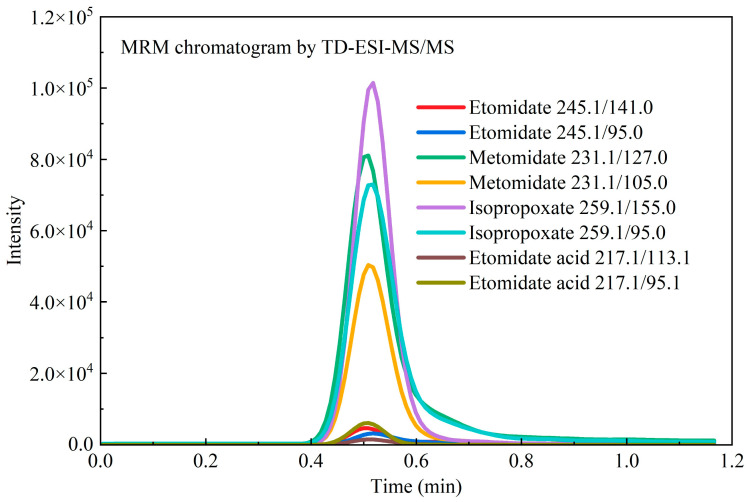
On-site MRM screening of a forensic hair sample using TD-ESI-MS/MS.

**Figure 2:**
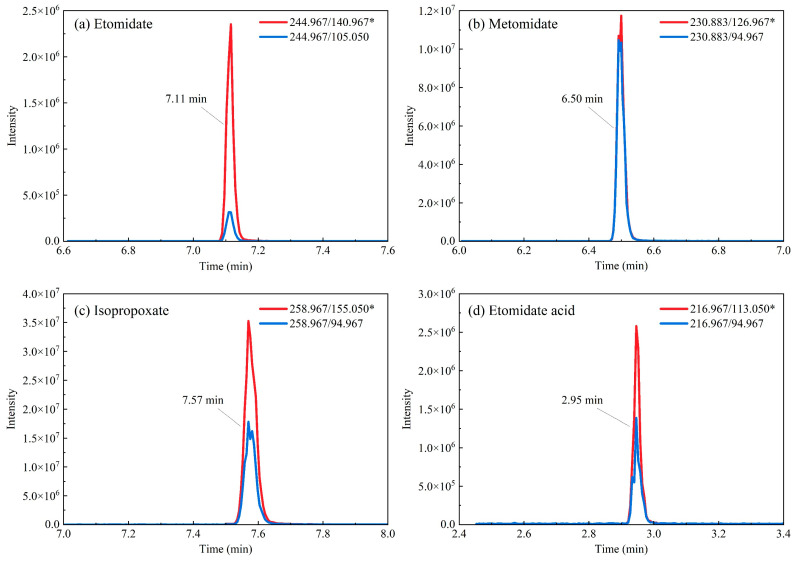
An MRM chromatogram of a forensic hair sample using UPLC-MS/MS. * represents quantified ion.

The integrated workflow was validated through an analysis of hair samples from a suspected polydrug user. Rapid screening using TD-ESI-MS/MS presumptively identified four etomidate analogs (ETO, METO, ISP, and ETA), with subsequent UPLC-MS/MS quantification confirming their concentrations at 0.5, 7.2, 11.3, and 0.4 ng/mg, respectively (Figure 1 and Figure 2). Etomidate, metomidate, and isopropoxate share structural alkyl tails (ethyl, methyl, and isopropyl/propyl) that might hydrolyze to release a common metabolite, etomidate acid. The detection of etomidate acid in the hair samples (Figure 1 and Figure 2) likely originated from one or more parent analogs prior to their metabolic conversion. This supports prior exposure to etomidate-derived NPSs in the investigated case, aligning with forensic toxicology reports documenting analogous metabolic pathways for structurally related sedatives [28].

#### 3.3.2. Large-Scale Performance Evaluation

**Figure 3:**
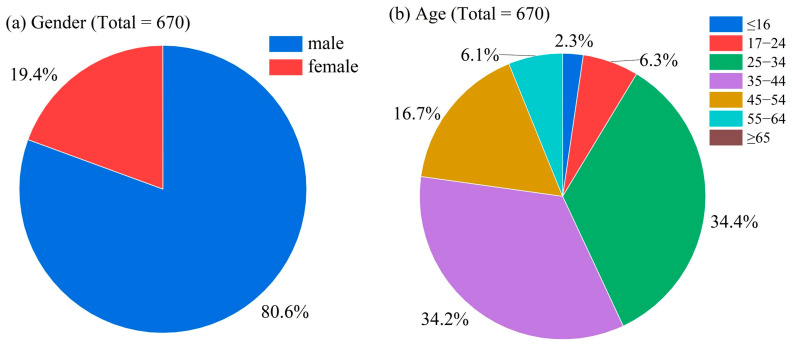
Subject demographics. A total of 670 hair samples were collected from drug rehabilitation centers and public security authorities. (**a**) Distribution of gender. (**b**) Distribution of age.

**Figure 4:**
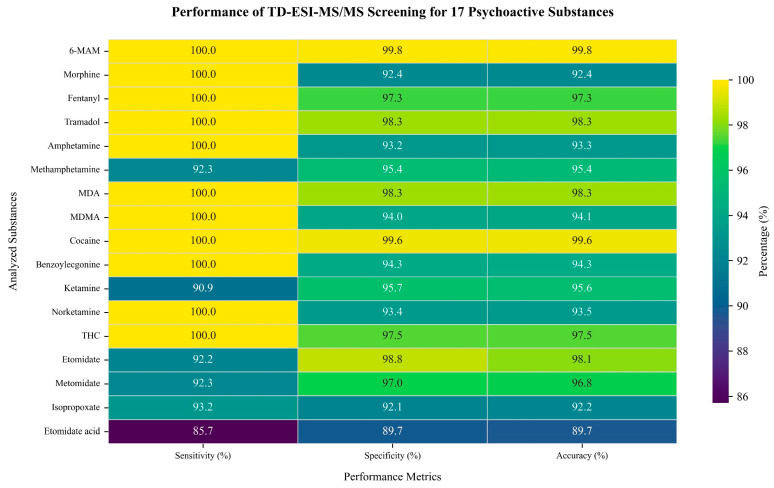
Heatmap of sensitivity, specificity, and accuracy for the detection of the 17 target analytes using TD-ESI-MS/MS.

The hair samples (*n* = 670) were obtained from drug rehabilitation centers and public security authorities. Self-reported demographic data collected via questionnaires revealed the predominance of male participants (80.6%). An analysis of their age distribution identified two predominant cohorts, 25–34 years (34.4%) and 35–44 years (34.2%), while only 8.6% represented individuals ≤ 24 years (Figure 3). A diagnostic performance evaluation of the TD-ESI-MS/MS method was conducted on the hair samples. Compared with the confirmatory results of UPLC-MS/MS, this method achieved ≥90.0% sensitivity and ≥90.0% specificity for 16 of the analytes while showing a slightly reduced performance for ETA (85.7% sensitivity, 89.7% specificity). The overall accuracy ranged from 89.7% to 99.8% (Figure 4), demonstrating < 10.3% combined false positive/negative rates. The TD-ESI-MS/MS method reduced the analysis time to 1 min/sample (vs. 10 min in LC-MS/MS) with ≥85.7% sensitivity, enabling 60 hair tests per hour shift to meet urgent surveillance throughput needs [29].

**Figure 5:**
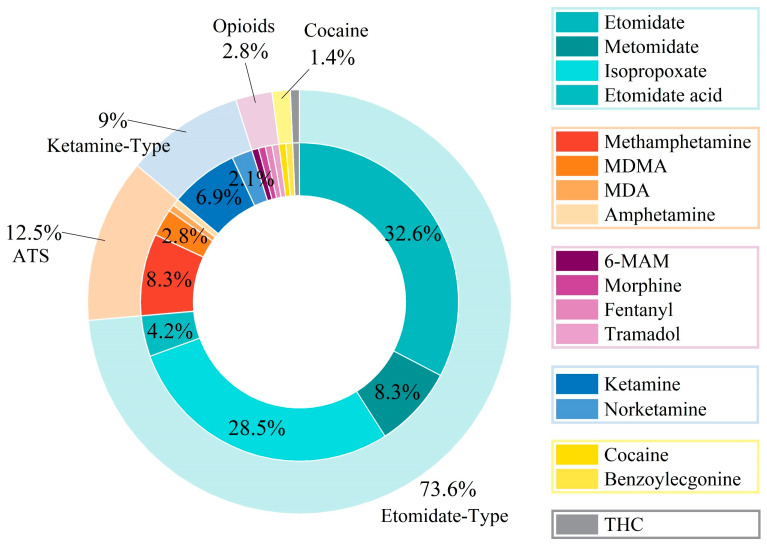
Distribution of the prevalence of six classes (opioids, ATS, cocaine, ketamine-type drugs, cannabinoids, and etomidate analogs) among 17 target analytes in the hair samples.

The analysis of specimens that were confirmed positive identified distinct trends in the prevalence of psychoactive substances (Figure 5). Etomidate analogs constituted the predominant chemical class (73.6%), with ETO (32.6%) and ISP (28.5%) as the primary components. ATS and ketamine-type drugs accounted for 12.5% and 9.0%, respectively, while opioids represented 2.8%. The relatively low ATS prevalence (12.5%) aligns with the success of interdiction programs targeting traditional illicit substances [29,30]. The high rate of etomidate analogs (73.6%) revealed key challenges in managing synthetic drugs. Effective control requires systematic tracking of new drug variants and rapid updates to the laws addressing chemically modified substances [31,32].

**Figure 6:**
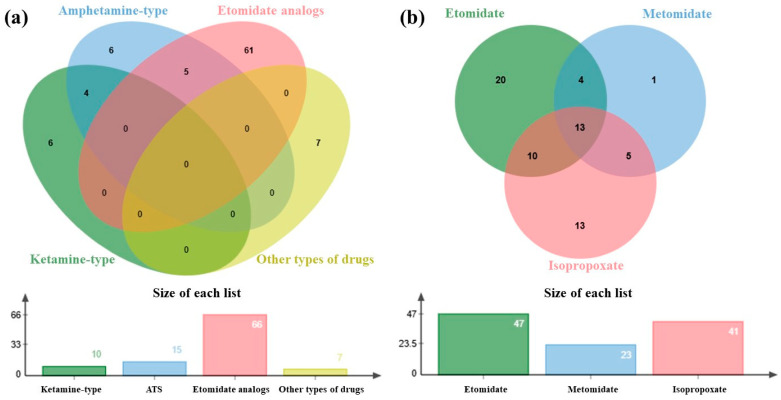
Venn diagram analysis of polydrug use patterns in forensic hair samples. (**a**) Cross-class co-occurrence of four major drug categories: ATS, ketamine-type drugs, etomidate analogs, and other drugs (cocaine, cannabinoids, and opioids). Overlapping regions represent samples with simultaneous detection of multiple classes. (**b**) Intra-class co-occurrence analysis of etomidate analogs (etomidate, metomidate, and isopropoxate).

The analysis of the polydrug use patterns revealed significant co-occurrence trends among the six drug categories (Figure 6). The co-occurrence analysis identified five cases of concurrent amphetamine–etomidate analog use and four cases of amphetamine–ketamine-type drug co-consumption. Notably, etomidate analogs exhibited the most complex polypharmacy profiles: 10 samples involved ETO paired with ISP, ETO was paired with METO in 4 samples, and 5 samples involved ISP-METO combinations. Strikingly, 13 samples demonstrated the simultaneous abuse of ETO, METO, and ISP, suggesting either shared procurement networks or escalating dependence patterns among users [33,34]. These findings suggest that the frequent co-administration of ATS with etomidate analogs (*n* = 5) may reflect evolving recreational drug combinations in high-risk populations, potentially driven by the synergistic effects of stimulants and dissociative anesthetics to amplify psychoactive experiences [35,36].

**Figure 7:**
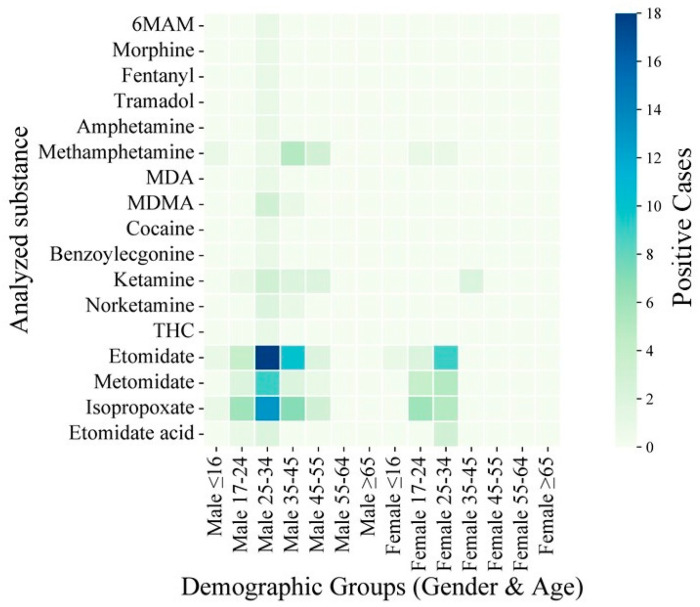
Age- and gender-stratified heatmap of heterogeneity of drug use.

The demographic heterogeneity in the drug use patterns was analyzed through a heatmap stratified by age and gender (Figure 7). The data revealed pronounced clustering of etomidate analogs among males aged 17–34, with 18 cases in males aged 25–34 and 10 cases in females aged 25–34. METH exhibited a bimodal distribution, peaking in males aged 35–45 (*n* = 5) and females aged 17–24 (*n* = 1). Ketamine-type drugs showed gender-specific disparities, with male predominance in the younger cohorts (17–24 years: *n* = 3) and sporadic female use in the older groups (35–45 years: *n* = 2). Strikingly, ISP, a structural analog of ETO, demonstrated the highest affinity with youth, with 6 cases in males aged 17–24 and 13 cases in males aged 25–34. These findings indicate the necessity of age- and gender-tailored intervention strategies. For instance, the concentration of etomidate analogs among young males warrants enhanced monitoring of e-commerce platforms and nightlife venues [37,38], while the bimodal METH distribution suggests the use of differentiated prevention campaigns targeting workplace stress management and risk education among adolescents [39,40].

### 3.4. Methodological Advantages and Forensic Implications

The integration of TD-ESI-MS/MS and UPLC-MS/MS established a rapid screening-to-confirmation workflow that significantly enhanced the operational efficiency for forensics. With a total analysis time of ≤20 min per sample (including ≤1 min for the TD-ESI-MS/MS screening), this approach enabled the high-throughput processing of large-scale casework, such as batch testing of hundreds of hair samples in a single forensic laboratory shift. TD-ESI-MS/MS demonstrated rapid screening capabilities in time-sensitive scenarios requiring immediate decision-making, such as monitoring drug abuse outbreaks. Cross-validation between TD-ESI-MS/MS and UPLC-MS/MS showed 89.7–99.8% accuracy. This reliability positions TD-ESI-MS/MS as a frontline screening tool for non-laboratory settings, complementing the traditional confirmatory methods without compromising the evidentiary standards. The predominance of etomidate analogs highlighted critical regulatory gaps in monitoring for emerging psychoactive substances. These findings align with China’s 2023 Schedule II reclassification of ETO, emphasizing the need for expanded toxicological surveillance networks targeting structural analogs.

### 3.5. Limitations of This Study

Hair offers unique advantages as a forensic matrix for analysis, including a prolonged detection window, non-invasive sampling, and the ability to reconstruct chronological drug exposure patterns via segmental analyses. However, limitations such as variability in hair growth rates, susceptibility to environmental contamination, and physicochemical alterations caused by cosmetic treatments must be critically addressed. Variations in the composition of hair, including its melanin content and keratin structure, between individuals may influence the incorporation of analytes. Further studies should employ keratin-adjusted normalization to enhance the quantitative comparability across populations. Furthermore, the long-term stability of polar analytes in stored hair samples remains contentious [41,42]. Participants who had undergone recent hair treatments (dyeing or bleaching within 6 months) were excluded from this study, while effects of residual cosmetics on the distribution of NPSs could not fully be discounted.

Additionally, the sampling strategy focused on high-risk populations, resulting in a positivity rate substantially higher than the community-level prevalence estimates. This selection bias limits the generalizability of the performance metrics to broader populations, such as workplace testing cohorts or asymptomatic users. To address these limitations, future studies should incorporate stratified sampling to evaluate the robustness of this method across diverse demographic and exposure scenarios.

TD-ESI-MS/MS demonstrated high specificity (>89.7% for 17 analytes), though the level of false positives remained legally critical due to the strict standards for the forensic evidence. The workflow addressed this limitation through mandatory UPLC-MS/MS confirmation. Potential matrix effects during screening necessitate strict quality control protocols in forensic practice.

The operational challenges associated with TD-ESI-MS/MS maintenance should also be paid attention to. Prolonged operation of the instrument (>8 h) led to observable signal attenuation (15–20% intensity loss), necessitating frequent ion source cleaning (every 500 samples). This maintenance requirement increases the downtime and consumable costs in high-throughput settings. While automated cleaning protocols mitigated these effects, further engineering optimizations, such as anti-fouling ion source designs or disposable emitter arrays, are needed to enhance its long-term stability for field deployments.

## 4. Conclusions

The integration of TD-ESI-MS/MS and UPLC-MS/MS established a reliable, high-throughput workflow for detecting multi-class drugs in hair samples, addressing critical needs in forensic toxicology and public health surveillance. The TD-ESI-MS/MS method’s ultra-rapid screening capability (1 min/sample) and high diagnostic accuracy (sensitivity > 85.7%, specificity > 89.7%) enabled efficient preliminary identification of drug exposure, particularly in time-sensitive scenarios. The confirmatory UPLC-MS/MS analysis ensured compliance with the validation standards.

Its large-scale application revealed the predominance of etomidate analogs (73.6% of positive cases), necessitating urgent updates to the international controlled substance lists and analog-specific legislation. Despite limitations in the temporal resolution and the representativeness of the cohort, this study demonstrated the viability of TD-ESI-MS/MS for bridging forensic and public health priorities. Future work should focus on optimizing the durability of the ion source for TD-ESI and validating this method across diverse populations to enhance its generalizability.

## Figures and Tables

**Table 1:** Validation results for the TD-ESI-MS/MS method.

Compound	LOD (ng/mg)	Concentration (ng/mg)	Inter-Day (*n* = 6)		Intra-Day (*n* = 6)		Matrix Effect (%)
			Accuracy (%)	Precision (%)	Accuracy (%)	Precision (%)	
6MAM	0.1	0.5	16.2	12.6	13.3	19.3	13.3
		2.5	6.8	6.2	9.8	12.1	9.0
		12.5	8.3	3.0	0.3	3.8	1.9
MOR	0.1	0.5	15.9	13.6	14.4	14.5	15.5
		2.5	5.6	7.1	8.8	2.7	3.6
		12.5	10.5	3.3	1.5	9.9	2.7
FENT	0.1	0.5	14.5	10.2	14.1	16.8	16.3
		2.5	6.2	12.4	1.6	7.7	9.7
		12.5	5.4	1.0	2.8	5.7	4.2
TRA	0.2	0.5	17.1	13.9	12.3	17.3	17.3
		2.5	8.0	5.8	6.3	1.6	2.2
		12.5	9.9	8.9	7.0	5.5	1.8
AMP	0.1	0.5	4.7	11.9	10.3	16.5	11.4
		2.5	7.6	1.2	4.3	7.0	3.1
		12.5	8.1	2.4	5.8	6.4	0.8
METH	0.1	0.5	12.2	15.8	13.7	14.8	10.2
		2.5	11.4	8.8	4.3	10.0	1.3
		12.5	6.8	4.1	9.5	4.7	3.2
MDA	0.2	0.5	12.7	15.4	12.1	17.1	12.5
		2.5	15.9	15.8	17.5	15.3	8.7
		12.5	6.9	8.0	3.9	5.3	7.7
MDMA	0.1	0.5	15.0	17.3	10.4	10.8	16.5
		2.5	3.4	1.8	16.1	7.1	3.1
		12.5	7.9	8.1	9.4	1.5	4.0
COC	0.1	0.5	3.1	3.0	5.2	9.9	9.9
		2.5	8.1	5.8	6.3	5.8	1.5
		12.5	5.1	4.1	1.3	6.8	2.1
BZE	0.1	0.5	10.7	8.4	16.5	18.3	10.8
		2.5	2.0	5.0	5.3	0.7	5.3
		12.5	9.8	0.6	0.7	1.4	3.6
KET	0.1	0.5	11.9	18.1	15.3	10.4	9.9
		2.5	7.0	5.4	10.4	9.2	7.5
		12.5	5.8	3.6	8.3	6.9	0.9
NorKET	0.1	0.5	12.5	11.3	10.8	18.8	8.1
		2.5	10.0	8.7	7.8	6.4	2.4
		12.5	4.9	0.4	1.9	0.1	5.2
THC	0.1	0.5	13.1	13.8	6.0	9.0	15.4
		2.5	11.7	8.6	17.8	16.8	8.9
		12.5	1.9	5.3	0.4	7.1	3.8
ETO	0.1	0.5	12.6	17.7	16.0	12.8	19.6
		2.5	1.3	6.9	3.9	2.6	5.5
		12.5	7.0	7.0	8.0	5.4	3.3
METO	0.1	0.5	16.4	10.1	15.6	8.0	14.4
		2.5	6.6	9.7	0.1	13.9	9.4
		12.5	0.5	7.1	2.4	8.4	7.1
ISP	0.1	0.5	11.8	10.4	14.1	17.7	19.6
		2.5	2.2	7.6	9.7	11.5	6.8
		12.5	4.5	9.6	6.7	6.9	5.6
ETA	0.2	0.5	19.0	15.5	14.5	15.0	18.3
		2.5	14.7	13.6	16.7	10.8	4.7
		12.5	4.3	2.7	5.6	2.1	5.8

**Table 2:** Validation results for the UPLC-MS/MS method.

Compound	LOD (ng/mg)	LLOQ (ng/mg)	Linearity and Range (ng/mg)	Correlation Coefficient	Concentration (ng/mg)	Inter-Day (*n* = 6)		Intra-Day (*n* = 6)	
						Accuracy (%)	Precision (%)	Accuracy (%)	Precision (%)
6MAM	0.01	0.02	0.02–2.50	0.9959	0.10	3.5	6.6	3.7	7.6
					1.00	5.5	3.9	6.7	5.1
					2.50	5.2	6.4	2.8	5.2
MOR	0.01	0.02	0.02–5.00	0.9988	0.10	8.1	6.1	6.8	4.8
					1.00	1.2	1.5	1.9	4.3
					2.50	7.6	8.5	4.4	1.6
FENT	0.01	0.02	0.02–5.00	0.9953	0.10	8.5	3.3	1.3	6.9
					1.00	1.3	2.0	5.3	1.5
					2.50	6.0	4.5	8.8	3.0
TRA	0.02	0.05	0.05–5.00	0.9962	0.10	2.0	8.5	6.1	8.5
					1.00	6.9	3.1	5.3	5.6
					2.50	7.2	1.7	8.0	1.2
AMP	0.01	0.02	0.02–5.00	0.9986	0.10	1.3	2.1	2.5	1.5
					1.00	8.1	5.2	5.8	2.4
					2.50	1.9	1.7	2.6	7.9
METH	0.01	0.02	0.02–5.00	0.9989	0.10	7.6	6.2	1.4	9.4
					1.00	8.1	5.2	3.7	5.4
					2.50	1.4	7.9	1.1	7.1
MDA	0.02	0.05	0.05–5.00	0.9958	0.10	5.3	6.6	5.0	5.7
					1.00	8.5	6.6	6.1	8.4
					2.50	2.2	6.2	6.9	3.6
MDMA	0.01	0.02	0.02–5.00	0.9960	0.10	6.1	2.7	7.5	5.7
					1.00	3.6	1.7	4.2	4.9
					2.50	1.8	5.4	8.4	8.1
COC	0.01	0.02	0.02–5.00	0.9991	0.10	6.9	8.0	8.8	4.9
					1.00	3.8	7.0	1.7	8.5
					2.50	8.9	4.2	8.5	6.0
BZE	0.01	0.02	0.02–5.00	0.9985	0.10	5.7	4.2	3.0	7.0
					1.00	6.2	8.3	3.4	3.4
					2.50	4.5	2.7	6.6	8.5
KET	0.01	0.02	0.02–5.00	0.9968	0.10	8.6	4.4	3.5	7.2
					1.00	7.4	6.5	2.3	4.0
					2.50	5.0	6.0	4.6	4.3
NorKET	0.01	0.02	0.02–5.00	0.9993	0.10	5.4	8.7	4.5	2.3
					1.00	3.6	7.4	6.5	4.4
					2.50	3.5	5.4	3.1	5.8
THC	0.01	0.02	0.02–5.00	0.9976	0.10	1.6	6.2	3.5	7.7
					1.00	8.2	8.5	5.8	6.3
					2.50	8.0	2.9	2.5	7.4
ETO	0.01	0.02	0.02–5.00	0.9959	0.10	4.6	5.0	7.3	6.1
					1.00	4.5	7.4	3.2	2.1
					2.50	7.1	6.7	2.1	2.3
METO	0.01	0.02	0.02–5.00	0.9985	0.10	5.7	4.8	4.1	7.4
					1.00	1.2	3.5	8.0	8.0
					2.50	5.8	7.8	6.3	7.2
ISP	0.01	0.02	0.02–5.00	0.9978	0.10	5.0	2.3	5.8	5.2
					1.00	3.5	4.6	5.4	3.3
					2.50	4.4	4.9	7.4	7.6
ETA	0.02	0.05	0.05–5.00	0.9973	0.10	2.8	4.7	8.6	3.1
					1.00	8.3	5.6	5.9	3.6
					2.50	7.9	7.0	4.9	4.9

## Data Availability

The original contributions presented in this study are included in the article. Further inquiries can be directed to the corresponding author(s).

## Supplementary Materials

The following supporting information can be downloaded at: https://www.mdpi.com/article/10.3390/toxics13050329/s1, Figure S1: MRM chromatogram of blank hair samples added with 2.5 ng/mg opioids (a), ATS (b), cocaine (c), ketamine-type (d), cannabinoids (e) and etomidate analogs (f); Table S1: Instrumental parameters for TD-ESI-MS/MS analysis; Table S2: Instrumental parameters for UPLC-MS/MS analysis; Table S3: Optimized parameters for TD-ESI-MS/MS analysis; Table S4: Optimized parameters for UPLC-MS/MS analysis.
