# Supplementary material for: TD-ESI-MS/MS for High-Throughput Screening of 13 Common Drugs and 4 Etomidate Analogs in Hair: Method Validation and Forensic Applications
Source: Toxics. 2025 Apr 23;13(5):329. doi: 10.3390/toxics13050329 (PMC12116117; doi:10.3390/toxics13050329)
Supplement: Supplementary file 1 [file toxics-13-00329-s001.zip › toxics-3569572-supplementary.pdf]

Supplementary Materials

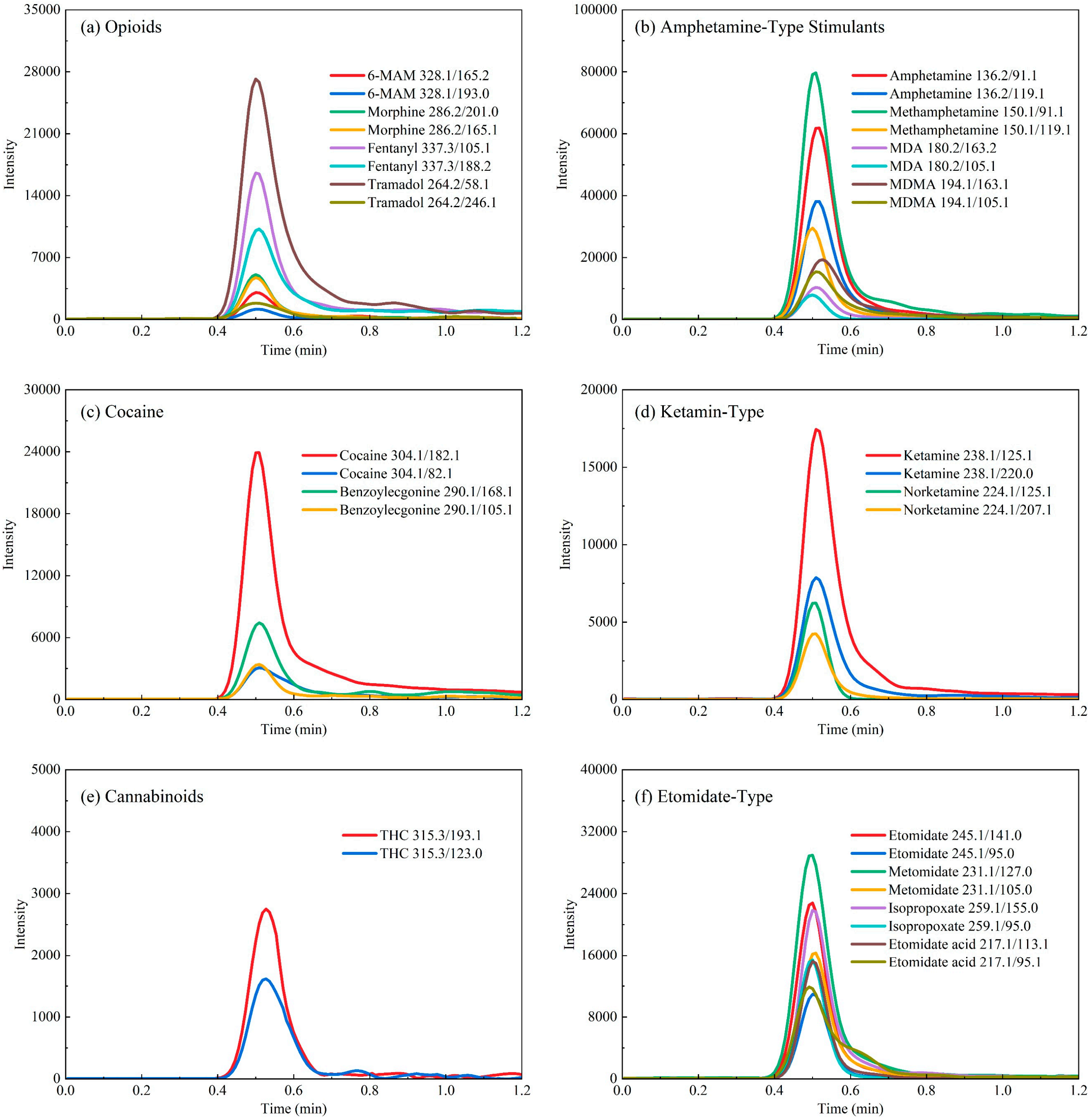

Figure S1. MRM chromatogram of blank hair samples added with 2.5 ng/mg opioids (a), ATS (b), cocaine (c), ketamine-type (d), cannabinoids (e) and etomidate analogs (f).

Table S1. Instrumental parameters for TD-ESI-MS/MS analysis.

Parameter	Setting/Value
Desorption temperature	260 °C
Capillary voltage	4 kV
Drying gas flow	3 L/min (300°C)
Atomizer pressure	5 psi
Solvent composition	0.1% formic acid in water<br>(containing 10 mmol/L ammonium formate-acetonitrile (1:1, v/v)
Solvent flow rate	200 μL/h

Table S2. Instrumental parameters for UPLC-MS/MS analysis.

Parameter	Setting/Value
Column	Shim-pack GIST C18-AQ (2.1 × 100 mm, 1.9 μm)
Column temperature	40 °C
Mobile phase gradient	0–0.5 min: 10% B<br>0.5–7.0 min: 10–95% B<br>7.0–8.5 min: 95% B<br>8.5–10.0 min: 10% B
Solvent composition	A: 0.1% formic acid in water; B: acetonitrile
Solvent flow rate	0.6 mL/min
Spray voltage	3200 V
Vaporizer temperature	350 °C
Collision energies	8.94-87.35 eV
Sheath gas	40 Arb
Aux gas	8 Arb
Sweep gas	1 Arb

Table S3. Optimized parameters for TD-ESI-MS/MS analysis.

Analyte	Precursor ion (*m*/*z*)	Product ion (*m*/*z*)	Fragmentor voltages (eV)	Collision energies (eV)	Polarity
6MAM	328.1	165.2	120	44	Positive
		193.0	120	32	Positive
MOR	286.2	201.1	190	28	Positive
		165.1	190	50	Positive
FENT	337.3	105.1	170	50	Positive
		188.2	170	24	Positive
TRA	264.2	58.1	130	28	Positive
		246.1	130	10	Positive
AMP	136.2	91.1	120	15	Positive
		119.1	120	5	Positive
METH	150.1	91.1	86	26	Positive
		119.1	86	10	Positive
MDA	180.2	163.2	180	6	Positive
		105.1	180	24	Positive
MDMA	194.1	163.1	120	12	Positive

		105.1	120	25	Positive
COC	304.1	182.1	124	18	Positive
		82.1	124	28	Positive
BZE	290.1	168.1	160	16	Positive
		105.1	160	30	Positive
KET	238.1	125.1	135	30	Positive
		220.0	135	14	Positive
NorKET	224.1	125.1	220	38	Positive
		207.1	220	12	Positive
THC	315.3	193.1	180	24	Positive
		123.0	180	32	Positive
ETO	245.1	141.0	100	5	Positive
		95.0	100	25	Positive
METO	231.1	127.0	80	4	Positive
		105.0	80	28	Positive
ISP	259.1	155.0	100	6	Positive
		95.0	100	28	Positive
ETA	217.1	113.1	50	5	Positive
		95.1	50	13	Positive

Table S4. Optimized parameters for UPLC-MS/MS analysis.

Compound	RT (min)	Precursor ion (*m*/*z*)	Product ion (*m*/*z*)
6MAM	3.21	328.050	165.050*, 211.133
MOR	1.10	286.050	165.133*, 201.133
FENT	5.60	337.103	188.133*, 105.050
TRA	4.42	264.050	58.050*, 42.217
AMP	3.15	135.967	90.967*, 119.050
METH	3.30	149.967	90.883*, 118.967
MDA	3.25	179.9	163.050*, 104.967
MDMA	3.40	193.967	163.050*, 134.967
COC	4.55	304.050	182.133*, 150.133
BZE	3.97	289.967	168.050*, 105.05
KET	3.71	237.983	124.883*, 219.967
NorKET	4.13	223.967	207.050*, 124.967
THC	9.55	315.133	193.133*, 259.217
ETO	7.11	244.967	140.967*, 105.050
METO	6.50	230.883	126.967*, 94.967
ISP	7.57	258.967	155.050*, 94.967
ETA	2.95	216.967	113.050*, 94.967

* represents quantified ion
